# Characterization of a Selenium-Tolerant Rhizosphere Strain from a Novel Se-Hyperaccumulating Plant *Cardamine hupingshanesis*

**DOI:** 10.1155/2014/108562

**Published:** 2014-11-12

**Authors:** Xinzhao Tong, Linxi Yuan, Lei Luo, Xuebin Yin

**Affiliations:** ^1^Advanced Lab for Selenium and Human Health, Suzhou Institute for Advanced Study, University of Science and Technology of China, Suzhou, Jiangsu 215123, China; ^2^Nano Science and Technology School, Suzhou Institute for Advanced Study, University of Science and Technology of China, Suzhou, Jiangsu 215123, China; ^3^Jiangsu Bio-Engineering Centre on Selenium, Suzhou, Jiangsu 215123, China; ^4^School of Earth and Space Sciences, University of Science and Technology of China, Hefei, Anhui 230026, China

## Abstract

A novel selenium- (Se-) hyperaccumulating plant,* Cardamine hupingshanesis*, accumulating Se as a form of SeCys_2_, was discovered in Enshi, Hubei, China, which could not be explained by present selenocysteine methyltransferase (SMT) theory. However, it is interesting to investigate if rhizosphere bacteria play some roles during SeCys_2_ accumulation. Here, one Se-tolerant rhizosphere strain,* Microbacterium oxydans*, was isolated from* C. hupingshanesis*. Phylogenetic analysis and 16S rRNA gene sequences determined the strain as a kind of Gram positive bacillus and belonged to the family* Brevibacterium frigoritolerans*. Furthermore, Se tolerance test indicated the strain could grow in extreme high Se level of 15.0 mg Se L^−1^. When exposed to 1.5 mg Se L^−1^, SeCys_2_ was the predominant Se species in the bacteria, consistent with the Se species in* C. hupingshanesis*. This coincidence might reveal that this strain played some positive effect in SeCys_2_ accumulation of* C. hupingshanesis*. Moreover, when exposed to 1.5 mg Se L^−1^ or 15.0 mg Se L^−1^, As absorption diminished in the logarithmic phase. In contrast, As absorption increased when exposed to 7.5 mg Se L^−1^, indicating As metabolism processes could be affected by Se on this strain. The present study provided a sight on the role of rhizosphere bacteria during Se accumulation for Se-hyperaccumulating plant.

## 1. Introduction

*Cardamine hupingshanesis* from the selenium (Se), mine drainage area in Enshi, China was identified as a new Se secondary accumulating plant, and it could accumulate up to 99% of total Se in the form of SeCys_2_ without showing any phytotoxic symptoms [1]. This plant is quite different with the ever known Se-hyperaccumulating plants,* Astragalus bisulcatus* with methylselenocysteine (MeSeCys) [2–5] and *γ*-glutamylmethylselenocysteine [2, 6], and* Stanleya pinnata* with MeSeCys [2, 7]. Presently, selenocysteine methyltransferase (SMT) was recognized as an important role on Se-hyperaccumulation because SMT could methylate SeCys to avoid its toxicity for related proteins [8, 9]. Moreover, the transgenic non-Se-hyperaccumulating plants,* Arabidopsis thaliana* and* Brassica juncea* (Indian mustard), overexpressing SMT could apparently accumulate Se in their tissues [10–12]. However, apparently, SMT theory could not give a reasonable explanation on SeCys_2_ hyperaccumulation in* Cardamine hupingshanesis* [1].

In recent years, some studies revealed that rhizosphere bacteria likely played an important role in facilitating the uptake of Se in Se hyperaccumulating plants [13, 14].* Astragalus bisulcatus* apparently lived with a variety of Se-resistant ecological partners (e.g., microbial endosymbionts) to affect Se species in plants [15, 16]. Rhizosphere bacteria increased Se enrichment and volatilization in* Brassica juncea* [16] and* Indian Mustard* [13] and similar phenomenon was also found in two wetland plants, saltmarsh bulrush and rabbit foot grass [13]. Moreover,* S. alfredii*, a kind of Cd/Zn hyperaccumulating plant, can tolerate higher metal concentrations with the assistance of bacteria [17]. The Ni-resistant rhizosphere bacteria could serve as an effective metal-immobilizing and growth-promoting bioinoculant for* N. caerulescens* [18]. Thus, the present study focused on the rhizosphere bacteria from* C. hupingshanesis*, and the special subjects are as follows: (1) Se-resistant bacteria were isolated from the rhizosphere of* C. hupingshanesis*; (2) one of them was choose to investigate the selenite metabolic process.

Furthermore, since high contents of arsenic (As) were also found in the sampling site (unpublished data, 17.78 ± 0.34 mg As kg^−1^) and As could affect the Se accumulation via antagonism or synergism effects [19], the relationship between Se and As in the selected strain's metabolic processes was also studied.

## 2. Materials and Methods

### 2.1. Study Site Description

**Figure 1:**
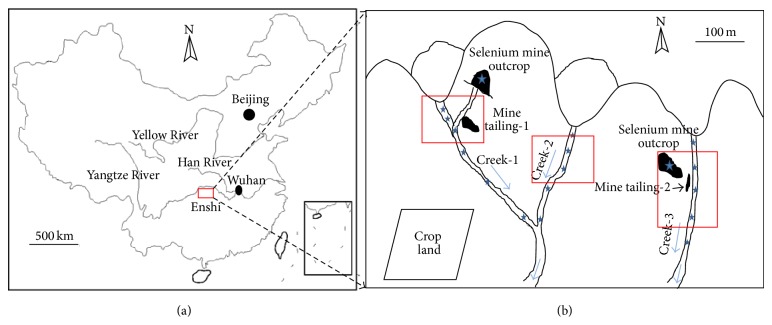
Study site and sampling: (a) the study area located in Enshi; (b) three sampling sites (red square) in selenium mine drainage areas.

The study site is located in Enshi, western Hubei province of China, and it was so-called as “World Capital of Se” because of the only Se mine in the world there.* Cardamine hupingshanesis*, a new Se secondary accumulator, was collected from Se-mine drainage area, Yutangba, northern Enshi (Figure 1). In general, the Se contents in sediments from the study site varied from 10 to 70 mg/kg DW with exceptional high near Se mine tailings (274 ± 152 (*n* = 3) mg/kg DW) [1]. High contents of As were also found in the rhizosphere soils of* C. hupingshanesis* (unpublished data, 17.78 ± 0.34 (*n* = 3) mg As kg^−1^).

### 2.2. Isolation of Rhizosphere Bacteria from* C. hupingshanesis*

Intact plants were dug out and their roots were chipped as 1-cm-long segments to macerate in 50 mL tube with sterile distilled water. After vibrating for 1 min, the homogenized suspensions were serially diluted to 10^−6^ times. Then, the diluted suspensions were plated on the surface of TSA medium with 200 *μ*g Se (as Na_2_SeO_3_)/L. Repeating the dilution and plating procedures till individual colony forming, the isolated rhizosphere strains were collected to store in glycerol stocks at −80°C.

### 2.3. Morphological, Physiological, and Biochemical Tests

One well-grown strain, labeled as Suzhou 08+6, was selected to do morphological, physiological, and biochemical tests. The light microscopy (Olympus BX51) was employed for morphological test. Physiological properties as well as enzyme activity tests and biochemical analysis (see Tables 2 and 3) were determined by the standard micromethod Api 20E. Those tests were performed in China Center for Type Culture Collection (CCTCC, Wuhan, China).

### 2.4. 16S rRNA Gene Sequencing

Genomic DNA was isolated from freshly grown culture (Suzhou 08+6) following the methods of Sambrook et al. (1989) [20]. The 16S rRNA gene was amplified using the universal primers 27f and 1492r [21], and the PCR products were sequenced by Invitrogen Biotechnology in CCTCC, Wuhan, China. The phylogenetic analysis was performed by using MEGA version 4.0 [22].

### 2.5. Total Se Analysis

The deposit (0.5–1 g) or supernatant (1 mL) samples after centrifuge were digested by HNO_3_ and HClO_4_ (4 : 1, v/v) for 12 h and then HCl (12 M) for 3-4 h according to Gao et al. (2011) [23]. The total Se concentrations were determined by Hydride Generation Atomic Fluorescence Spectrometry (HG-AFS 9230) (Beijing Titan Instrument Co., China) with 0.75% RSD.

### 2.6. Total As Analysis

The deposit (0.5–1 g) or supernatant (1 mL) samples after centrifuge were digested by HNO_3_ and HClO_4_ (4 : 1, v/v) for 12 h and then VC-thiourea (50 g L^−1^) for 2 h according to Bai et al. (2009) [24]. The total As concentrations were determined by Hydride Generation Atomic Fluorescence Spectrometry (HG-AFS 9230) (Beijing Titan Instrument Co., China) with 1.37% RSD.

### 2.7. Se Speciations Analysis

The deposition (0.2 g) or supernatant (1 mL) samples were attracted in Tris-HCl buffer (100 mM, pH 7.5) and proteinase (100 mM) for 14 h at 50°C and then protease XIV for 8 h at 37°C according to Liang et al. (2006) [25] and Mazej et al. (2006) [26]. The Se speciations were determined by liquid chromatography-UV irradiation-hydride generation-atomic fluorescence spectrometry (LC-UV-HG-AFS).

### 2.8. Growth Curve of Bacteria

Absorbance value of bacterial culture was determined at *λ* = 600 nm by Ultraviolet-Visible Pectrophotometer (UV-2450, Shimadzu) at different time intervals.

### 2.9. Design for Se and As Interaction Observations

Seven experimental groups and one control group were designed to observe the interactions between Se and As. The control group was added the isolated strain Suzhou 08+6 without Se or As. Three of the experimental groups were added with three different levels of Se (as selenite) (1.5, 7.5, and 15.0 mg L^−1^) but no As. One of the experimental groups were only added with As (as sodium arsenate) as 0.5 mg L^−1^, and the other three experimental groups were added with three different levels of Se (1.5, 7.5, and 15.0 mg L^−1^) and one level of As (0.5 mg L^−1^) (Table 1).

## 3. Results and Discussion

### 3.1. Identification of Rhizosphere Strain from* Cardamine hupingshanesis*

**Figure 2:**
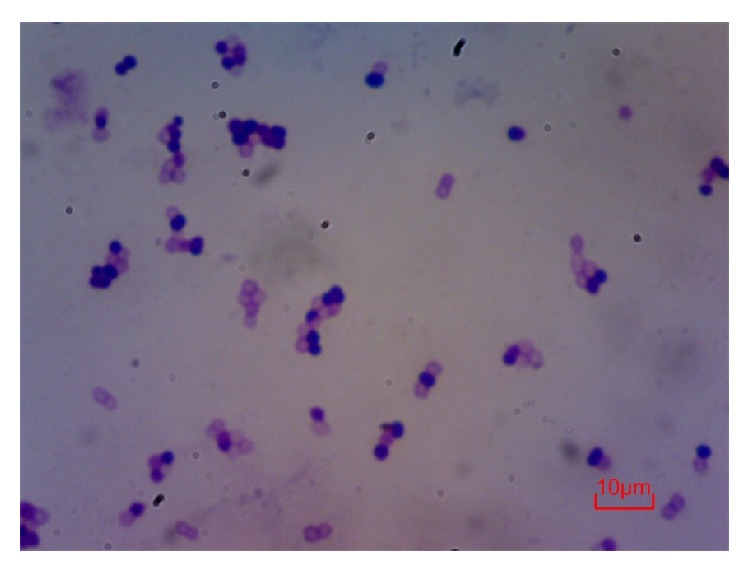
Cell morphology of isolated rhizospheric strain, Suzhou 08+6, under microscopy ×1000 (Gram staining method).

After successively culturing in 200 Se *μ*g/L mediums, 11 strains were collected, and one of them, named as Suzhou 08+6, was selected to be performed morphological, physiological, and biochemical traits. The observation under light microscopy displayed the isolated strain, Suzhou 08+6, could be a kind of Gram positive bacillus (Figure 2).

**Figure 3:**
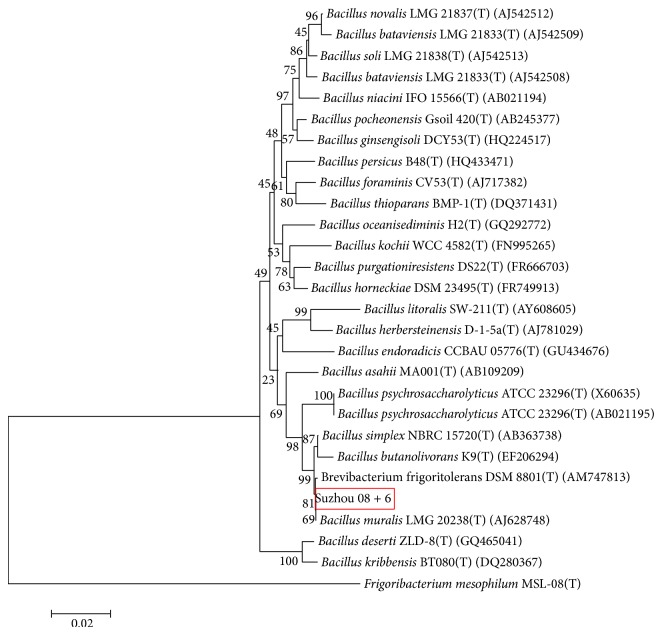
Phylogenetic positions based on neighbour-joining of the 16S rRNA gene sequence of the isolated rhizosphere strain, Suzhou 08+6.

16S rRNA phylogenetic tree analysis revealed that Suzhou 08+6 displayed a high similarity (99.93%) to* Brevibacterium frigoritolerans* (GeneBank accession number DSM 8801(T)) (Figure 3).* B. frigoritolerans*, originally isolated from the arid soils of Morocco by Delaporte and Sasson (1967) [27], was a strictly aerobic chemoorganotrophic member of the genus* Brevibacterium* and had a unique feature on cold-resistance.

The physiological and biochemical analysis showed that Suzhou 08+6 could grow in 2% or 5% NaCl mediums and produce a series of active enzymes, including esterase (C4), esterase lipase (C8), naphthol-AS-BI-phosphate hydrolase, *β*-galactosidase, *α*-glucosidase, and *β*-glucosidase (Table 2). In addition, the mannitol and amygdalin could be used as carbon sources by Suzhou 08+6 (Table 3).

Combined with above results, the isolated strain Suzhou 08+6 was identified as* Microbacterium oxydans*.

### 3.2. Selenium Metabolic Characteristics of the Isolated Strain Suzhou 08+6

**Figure 4:**
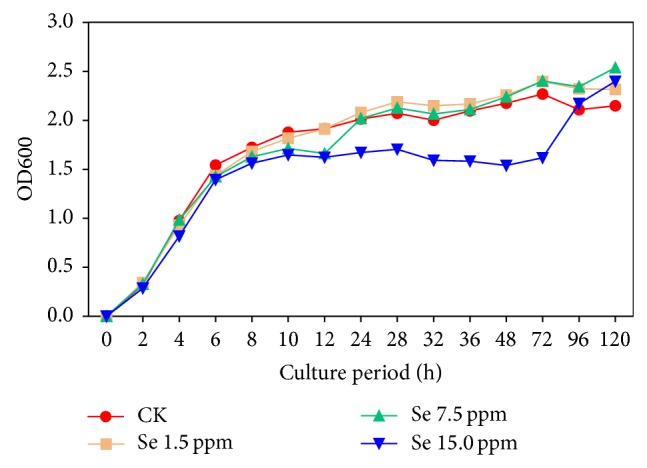
Growth curves of isolated strain in different Se medium. CK: growth in the culture without selenite; Se 1.5: grow in the culture with 1.5 mg Se L^−1^; Se 7.5: grow in the culture with 7.5 mg Se L^−1^; Se 15.0: grow in the culture with 15 mg Se L^−1^ (*n* = 5).

To investigate the tolerant Se-levels of Suzhou 08+6 (*Microbacterium oxydans)*, the strain was grown in liquid TSB with 0, 1.5, 7.5, and 15.0 mg Se L^−1^, respectively, and the kinetics of bacteria growth was traced by OD 600 in the cultures. The results (Figure 4) showed that Suzhou 08+6 could normally grow in those four different cultures, even in the extreme high level of 15.0 mg Se L^−1^, indicating the strain could be a sort of Se-tolerant bacterium. The isolated strain was characterized by logarithmic phase in first 8 h and stationary phase in the following 8–120 h. Overall, there were no obvious differences among CK, Se 1.5, and Se 7.5. However, the absorbance values in 15.0 mg Se L^−1^ group were 30% less than those in the other three groups, especially during stationary phase, suggesting that Se concentrations in the medium (>7.5 mg Se L^−1^) are likely the limiting factor for Suzhou 08+6 growth. Tetteh et al. (2014) revealed that 5 mM Na_2_SeO_3_ treatment inhibited* E. coli* growth by 50%, whereas 0.001 to 0.01 mM Na_2_SeO_3_ treatments stimulated cell growth by 30%. In addition, cell numbers drastically decreased when treated with 50 mM or higher Na_2_SeO_3_ [28, 29]. This phenomenon could be attributed to the interactions between Se and enzymes [30, 31], and Se probably exerts inhibiting effect on the activity of enzymes with the catalysis oxidation reactions of S–H groups to S–S or S–Se–S bonds [32].

Suzhou 08+6 was cultured in liquid TSB with 1.5 mg Se L^−1^ to observe its Se metabolic process.

**Figure 5:**
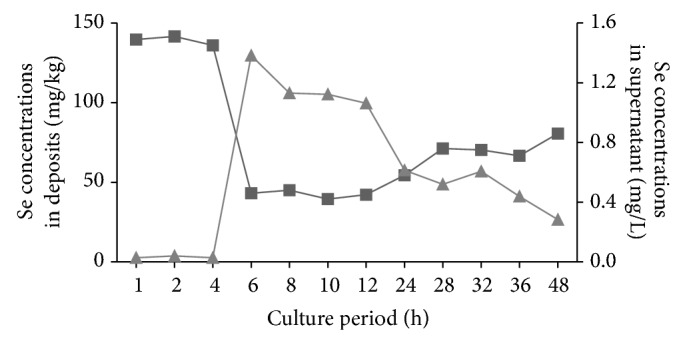
Se concentrations in the supernatant and deposit. ■: Se concentrations in supernatant (mg L^−1^); ▲: Se concentrations in deposit (mg kg^−1^).

HG-AFS and LC-UV-HG-AFS analysis indicated that Se (IV) in culture medium was actively consumed by bacteria in exponential phase, and SeMeCys (1.77 mg kg^−1^) was detected in deposit at 4 h (Table 4). After six hours, selenite contents diminished by 66% of the initial value in the supernatant, and Se achieved at a maximum concentration (130 mg Se kg^−1^) in the deposits (Figure 5). The predominant Se species in the deposits was SeCys_2_, which was accounted for more than 80% of total Se during the culture period. Moreover, SeMeCys and Se (IV) were also detected in bacterial deposit. In contrast, the primary Se species in supernatant was Se (IV) and the content of total Se was decreased gradually in exponential phase (Table 4). However, as the strain growth entered into stationary phase, selenite contents in the supernatant increased by 30%, which might be attributed to the Se absorption reduced 70% in bacteria (Figure 4). The contents of Se species such as SeCys_2_ in the deposits decreased steadily, from 14.09 mg kg^−1^ at 6 h to 2.64 mg kg^−1^ at 48 h. Thus, the isolated bacterium, Suzhou 08+6 (*Microbacterium oxydans)*, could transform Se (IV) into SeCys_2_, which was consistent with the dominant Se speciation accumulated in* C. hupingshanesis* [1].

### 3.3. Interactions between Se and As in the Metabolic Process of the Isolated Strain

**Figure 6:**
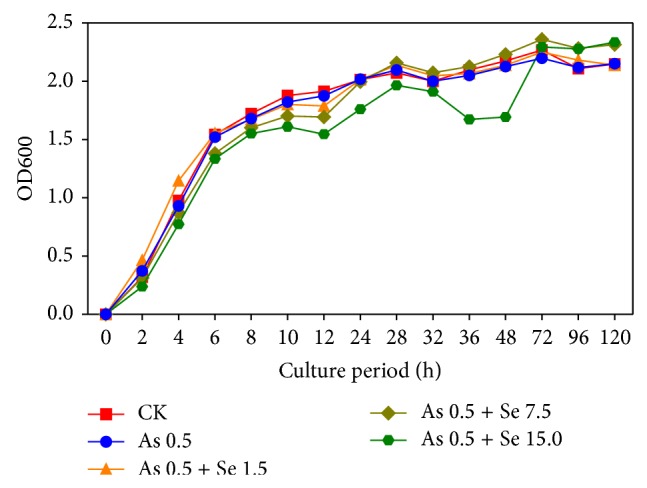
The growth of the isolated strain, Suzhou 08+6, in the culture medium with different concentration of Se and As. One control group and four experimental groups with 0.5 mg As L^−1^ and different concentration of Se (0, 1.5 mg Se L^−1^, 7.5 mg Se L^−1^, and 15.0 mg Se L^−1^) in the culture medium (*n* = 5).

To study the interactions between Se and As, Suzhou 08+6 was grown in liquid TSB with different concentrations of selenite and arsenic (Table 1). The results displayed that Suzhou 08+6 could grow in five different culture mediums (Figure 6). Overall, there were no obvious differences among CK, As 0.5 + Se 1.5, and As 0.5 + Se 7.5. However, high concentration of selenite (15.0 mg Se L^−1^) might have a strong inhibited effect on strain growth during the culture period in As 0.5 + Se 15.0 group.

**Figure 7:**
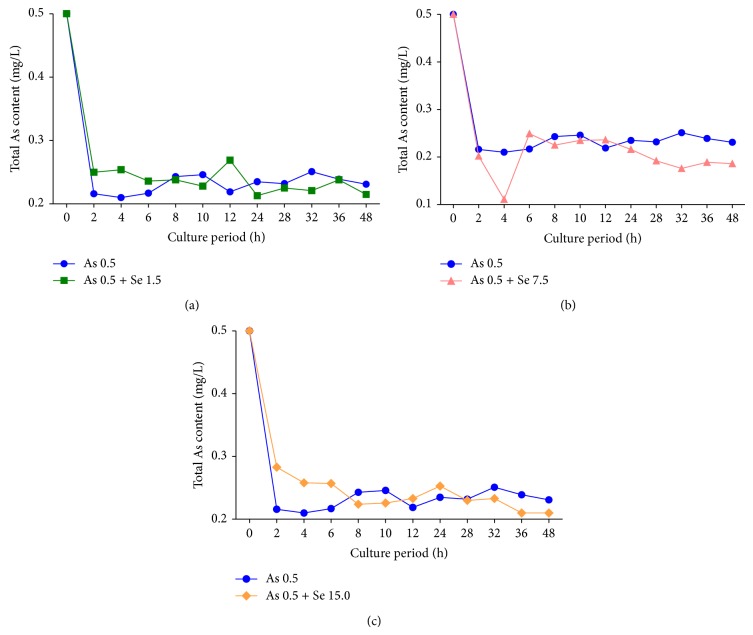
The content of total As in supernatant of different experimental groups. (a) As 0.5, As 0.5 + Se 1.5 (b) As 0.5, As 0.5 + Se 7.5 (c) As 0.5, and As 0.5 + Se 15.0.

HG-AFS analysis showed that total As in the supernatant was increased about 30% in the logarithmic phase with the presence of 1.5 mg Se L^−1^ (Figure 7(a)) and 15.0 mg Se L^−1^ (Figure 7(c)), indicating As absorption in the strain decreased under Se stress in these two groups. However, total As in supernatant diminished by 50% during logarithmic phase and stationary phase with the presence of 7.5 mg Se L^−1^ (Figure 7(b)), indicating As absorption in the strain was increased under Se stress in this group.

The above results indicated that the interactions between Se and As on Suzhou 08+6 depended on Se and As levels in culture mediums. Under arsenic stress (0.5 mg As L^−1^), Se could inhibit As absorption at the level of below 1.5 mg Se L^−1^ or above 15.0 mg Se L^−1^. But the strain growth would be strongly inhibited at the level of 15.0 mg Se L^−1^, compared with no inhibition at the level of 1.50 mg Se L^−1^ Interestingly, Se could promote As absorption at the moderate levels (e.g., 7.5 mg Se L^−1^), without showing obvious effect on strain growth.

Zhou et al. (1987) revealed that 5 mg L^−1^ or lower Se might antagonize As absorption, whereas 10 mg L^−1^ Se could synergize As absorption on* Photobacterium Phosphoreum* T3 [33]. When* E. coli* was exposed to 89 g L^−1^ or 178 g L^−1^ selenomorpholine, Se significantly antagonized As absorption. However, when exposed to a higher selenomorpholine (446 g L^−1^), Se only has antagonistic effects on As absorption with higher As (6–8 g L^−1^) and has cooperative interaction with lower As (1–4 g L^−1^) [19].

## 4. Conclusions

One Se-tolerant rhizosphere strain,* Microbacterium oxydans*, was isolated from* Cardamine hupingshanesis* and identified as a family of* Brevibacterium frigoritolerans* by 16S rRNA phylogenetic analysis. Se tolerance test indicated that the isolated strain could grow in extreme high Se level of 15.0 mg Se L^−1^. When exposed to 1.5 mg Se L^−1^, SeCys_2_ was the predominant Se species in the bacterial deposits, which is coincided with the dominant Se species in* C. hupingshanesis*, indicating this kind of Se-tolerant strain played some positive effects in SeCys_2_ accumulation of* C. hupingshanesis*. Moreover, Se and As interactions on this isolated strain were also studied. When exposed to 1.5 mg Se L^−1^ or 15.0 mg Se L^−1^, As absorption diminished by 20–30% in the logarithmic phase. In contrast, As absorption increased when exposed to 7.5 mg Se L^−1^.

## Figures and Tables

**Table 1:** Experimental design of the metabolic process of the isolated bacteria on Se and As.

	No Se	Low-level Se (1.5 mg L^−1^)	Middle-level Se (7.5 mg L^−1^)	High-level Se (15 mg L^−1^)
No As	CK	Se 1.5	Se 7.5	Se 15.0
As (0.5 mg L^−1^)	As 0.5	Se 1.5 + As 0.5	Se 7.5 + As 0.5	Se 15.0 + As 0.5

**Table 2:** The growth characteristics and enzyme activity of the isolated strain Suzhou 08+6.

Characteristics	Results	Characteristics	Results
Growth on 2%, 5% NaCl	+	Acid phosphatase (ACP)	−
Amylase	−	Naphthol-AS-BI-phosphate hydrolase	+
Alkaline phosphatase	−	*α*-Galactosidase	−
Esterase (C4)	+	*β*-Galactosidase	+
Esterase lipase (C8)	+	*β*-Glucuronidase	−
Lipase (C14)	−	*α*-Glucosidase	+
Leucine arylamidase	−	*β*-Glucosidase	+
Valine arylamidase	−	N-Acetyl glucosamine-enzyme	−
Cystine arylamidase	−	*α*-Mannosidase	−
Trypsin	−	*β*-Fucosidase	−
Chymotrypsin	−		

+: presence of a trait; −: absence of a trait.

**Table 3:** API 20 enzymes and carbon source fermentation on the isolated strain Suzhou 08+6.

Characteristics	Results	Characteristics	Results
*β*-Galactosidase	−	Gelatinase	−
Arginine hydrolase	+	Glucose fermentation/oxidation	−
Lysine decarboxylase	−	Mannitol Fermentation/oxidation	+
Ornithine decarboxylase	−	Inositol fermentation/oxidation	−
The use of citric acid	−	Sorbitol fermentation/oxidation	−
H_2_S production	−	Rhamnose fermentation/oxidation	−
Urease	−	sucrose fermentation/oxidation	−
Tryptophan deaminase	+	Melibiose fermentation/oxidation	−
Indole production	−	Amygdalin fermentation/oxidation	+
Acetoin production	+	Arabinose fermentation/oxidation	−

+: presence of a trait; −: absence of a trait.

**Table 4:** Concentrations of Se speciations (Se^4+^, SeCys_2_, SeMecys, and SeMet) in the supernatant and deposit. (The isolated strain, Suzhou 08+6, was grown in the culture medium with 1.5 mg Se L^−1^).

Time	Se speciation concentrations in supernatant (mg L^−1^)				Se speciation concentrations in deposit (mg kg^−1^)			
	Se^4+^	SeCys_2_	SeMeCys	SeMet	Se^4+^	SeCys_2_	SeMeCys	SeMet
1 h	1.54	—	—	—	∗	∗	∗	∗
2 h	1.57	—	—	0.02	∗	∗	∗	∗
4 h	1.52	—	—	—	—	—	1.77	—
6 h	0.03	—	—	—	0.37	14.09	—	—
8 h	0.08	—	—	—	—	22.20	—	—
10 h	0.06	—	—	—	—	12.26	3.54	—
12 h	0.07	—	—	—	—	22.05	—	—
24 h	0.07	—	—	—	0.19	7.06	—	—
28 h	0.11	—	—	—	0.10	12.19	—	—
32 h	0.08	—	—	—	0.20	11.92	—	—
36 h	0.07	—	—	—	0.23	7.42	—	—
48 h	0.07	—	—	—	—	2.64	—	—

—: below the detection limit; ∗: no sample.
